# Supplementary material for: Spurious regulatory connections dictate the expression‐fitness landscape of translation factors
Source: Mol Syst Biol. 2021 Apr 26;17(4):e10302. doi: 10.15252/msb.202110302 (PMC8073009; doi:10.15252/msb.202110302)
Supplement: Supplementary file 1 — Appendix [file MSB-17-e10302-s007.pdf]

Appendix Table of Contents

APPENDIX SUPPLEMENTARY METHODS *2*

Stochastic queuing model of ribosomes **2**

Predictions of bacterial growth laws for gratuitous protein expression **2**

Tuning PrmC under RF2 overexpression **3**

RF1 knockdown and global changes in expression **3**

Bacterial growth laws **4**

APPENDIX FIGURES *6*

APPENDIX SUPPLEMENTARY METHODS

Stochastic queuing model of ribosomes

The number of ribosomes on an mRNA can be approximated by solving a totally asymmetric exclusion process (Shaw *et al*, 2003), but a further simplified model disregarding spatial information recapitulates the statistics of queue formation (as verified by full stochastic simulations, data not shown). The state space of the simplified queue model is the number of ribosomes N in the queue upstream of the stop codon (where $N = 1$ when one ribosome is on the stop codon). Ribosomes arrive at a rate α (initiation rate on the transcript), and leave at the termination rate β. The ribosome arrival rate at the queue is rigorously equal to α in steady-state (we neglect mRNA degradation in this discussion), unless the queue becomes large enough to affect the initiation process (fully jammed transcript). This jammed regime is far from considered conditions, even with severe RF depletion (as determined from the typical queue length from our metagene analysis, Fig. 4A)

The stochastic process away from the jammed state is then described by: $N \to N + 1$ at rate α (ribosome arriving at the stop codon), and $N \to N - 1$ at rate β (ribosome leaving the stop codon) for $N > 0$. It assumes that the processes of initiation and termination are independent of the number of ribosomes in the queue. The probability for the queue to have N ribosomes, $P(N)$, can be obtained by solving the steady-state solution of the resulting master equation, leading to a geometric series: $P(N) = (\alpha/\beta)^N(1 - \alpha/\beta)$. Hence, the prevalence of higher order queues scales as the ratio of the initiation to termination rate on the transcript to the power of the number of ribosomes queued. The average queue size, i.e., $\langle N \rangle$, is $\alpha/(\beta - \alpha)$. The divergence of queue length as initiation rate α approaches termination rate β corresponds to the jamming transition, with long queues. This relationship shows that upon decrease of termination rate (e.g., from RF depletion), mRNAs with larger initiation rate (translation efficiencies), are expect to have longer queues (schematically illustrated in Fig. EV5H), which is what we observe on individual genes (Fig. EV5B-C).

Predictions of bacterial growth laws for gratuitous protein expression

The *E. coli* bacterial growth laws (section "Bacterial growth laws" below) formulate predictions of the impact of gratuitous expression on the translation sector compression and growth. Coarse-grained regulatory parameters ($\phi_\circ, \phi_R^{max}, \kappa_n, \kappa_t$) required for the predictions can be obtained from fits of physiological trajectories in the space of growth rate vs. translation sector space (Fig. 2J-L). The resulting predictions (Eq. 1 and 2) with parameters obtained here are shown as dashed lines in Fig. 2J-L.

Central to the *E. coli* growth law predictions is the incompressibility of a large portion (Q sector in (Scott *et al*, 2010)) of the proteome, which leads to steeper growth defect upon gratuitous expression. In contrast, in our comparison with the estimated proteome fraction occupied by the $\boldsymbol{\sigma}^B$ regulon, we find that growth defect is well explained by a purely passive compression factor of $1 - \phi_U$ (no incompressible sector, full lines in Fig. 3E, 3H, EV3G, EV3I), c.f. Eq. 1 and 2 below. Incompressibility was hypothesized to arise from autoregulatory mechanisms, which could be species specific and not be present in *B. subtilis*.

Tuning PrmC under RF2 overexpression

In order to verify that the growth defect resulting from RF2 overexpression ($s \approx -6\%$, Fig. 2F) was not caused by imbalance between RF and PrmC, we tuned PrmC over the range achievable with our inducible construct under maximal RF2 overexpression (Fig. 2D, 2H). Increased PrmC expression above wild-type level did not measurably rescue growth, suggesting that the growth defect for RF2 overexpression was not due to lack of post-translational modification of RF2 resulting from insufficient PrmC levels.

Consistently with the observations for wild-type RF2 levels, over-expressing PrmC led to a large induction in the $\boldsymbol{\sigma}^B$ regulon (Fig. EV3C), with associated translation sector compression (Fig. EV3G), and growth defect (Fig. EV3H). Transcriptomic re-arrangements following PrmC overexpression were highly reproducible and largely independent of RF2 levels (Fig. EV3J).

In contrast to the lack of change upon PrmC overexpression, the growth defect observed for PrmC knockdown was aggravated by RF2 overexpression (Fig. EV3M-N), possibly due to increased stoichiometric imbalance between PrmC and the total RF1 and RF2 concentration. Although mild $\boldsymbol{\sigma}^B$ induction was observed in these conditions (Fig. EV3A, EV3E), this induction was not causative of the observed growth defect, as deleting *sigB* did not rescue growth (blue shading, Fig. EV3E-F, EV3H) in this region of the RF expression subspace. In particular, the translation sector was compressed, seemingly because of large-scale transcriptomic changes independent of *sigB* (Fig. EV3A-B and insets), in contrast to the situation for overexpression of PrmC. The identification of the events leading to these transcriptional changes will warrant further studies.

RF1 knockdown and global changes in expression

Upon steady-state RF1 knockdown, and associated with the sharp decrease in cell growth (Fig. 2E, EV4B), we observe large changes in specific regulons (some examples shown in Fig. EV4C), analogously to $\boldsymbol{\sigma}^B$ induction upon PrmC overexpression. For example, members of the motility regulon (driven by alternative sigma factor $\boldsymbol{\sigma}^D$, gene *sigD*) and *lyt* operon, have a >5× median decrease in mRNA levels, while mRNA levels of biofilm production matrix genes (*eps* operon) are up about 10× (Fig. EV4D). These effects are independent of *sigB* (Fig. EV4E).

Attempting to identify causative molecular events responsible for these changes is complicated by the large number of regulators controlling motility in *B. subtilis* (Mukherjee & Kearns, 2014). Our CRISPRi depletion of RF1/PrmC, which was not in steady-state of growth, indicates that induction of *eps* genes are likely initial steps in the cascade, as their expression is up before large changes to $\boldsymbol{\sigma}^D$ and *lyt* operons (median fold-change increase of 2.9 compared to wild-type for the data in Fig. 4C, not highlighted on figure). Multiple regulators are known to modulate expression of *eps* genes (e.g., RemA (Winkelman *et al*, 2013), SinR (Kearns *et al*, 2005), and DegU (Murray *et al*, 2009)), and many have connections to RF1. *remA* is an operon with an upstream gene with the RF1 UAG stop codon, although the two genes are separated by a large distance (77 bp) and show no changes in expression stoichiometry upon RF1/PrmC depletion in our dataset (not shown). Both *sinR* and *degU* end with the RF1 UAG stop. These provide plausible candidate stop codons to modify for attempting to rewire of the RF1 landscape, analogously to our modifications of the $\boldsymbol{\sigma}^B$ operon (Fig. 5), although identification of the regulatory network's focal point in this instance lies beyond the scope of the current work.

Bacterial growth laws

The bacterial growth laws (Scott et al., 2010, 2014; You et al., 2013) constitutes an empirical framework recapitulating the global regulatory architecture of *E. coli* under nutrition and translation stress in steady-state growth. The growth laws connect the growth rate λ to the abundance of coarse-grained proteome sectors (translation or "ribosome affiliated" proteins ϕ_R; unregulated proteins which includes metabolic enzymes ϕ_P, and gratuitous unnecessary non-toxic proteins ϕ_U) through three mathematical relationships:

$$\begin{aligned} \text{Resource allocation: } \phi_R^{max} &= \phi_U + \phi_R + \phi_P, \\ \text{Nutrient flux: } \rho\lambda &= \kappa_n \phi_P, \\ \text{Protein synthesis: } \rho\lambda &= \kappa_t(\phi_R - \phi_\circ). \end{aligned}$$

Above, phenomenological parameters $\phi_\circ$ corresponds to an inactive ribosome fraction, κ_n is the nutritional capacity, κ_t is the translational capacity (proportional to the rate of protein synthesis), and ρ is a fixed conversion factor. Nutritional and translational capacities are defined through slopes of the lines along physiological trajectories.

The growth laws can be interpreted as amino acid flux conservation in steady-state growth: the flux generated by catabolic enzymes and transporters (ϕ_P) matches the biomass production flux by translation proteins (ϕ_R).

Changing the medium quality changes nutritional capacity κ_n without altering the translational κ_t and inactive ribosome content $\phi_\circ$. Then, the translation sector ϕ_R decreases following the nutrition line:

$$\text{Nutrition line: } \phi_R = \phi_\circ + \frac{\lambda}{\kappa_t}.$$

In contrast, in a fixed medium (fixed κ_n), but upon perturbation to translation (either through the increasing the fraction of inactive ribosome $\phi_\circ$, e.g., by chloramphenicol treatment (Dai et al., 2016; Scott et al., 2010), or decreasing translation rate κ_t, e.g., by fusidic acid treatment (Zhu *et al*, 2019)), the translation sector increases following:

$$\text{Translation line: } \phi_R = \phi_R^{max} - \frac{\lambda}{\kappa_n}.$$

Upon expression of unnecessary proteins to proteome fraction ϕ_U, the growth laws above predict movement along the nutrition line (since κ_t and $\phi_\circ$ are assumed fixed), and a fractional decrease in the translation sector and growth:

$$\frac{\phi_R}{\phi_R^\circ} = 1 - \frac{\phi_U}{\phi_R^{max} + \frac{\kappa_t \phi_\circ}{\kappa_n}}, \quad Eq.1$$

$$\frac{\lambda}{\lambda^\circ} = 1 - \frac{\phi_U}{\phi_R^{max} - \phi_\circ}. \quad Eq.2$$

The slope of ϕ_R vs. $\phi_U$is determined by, which relates to the posited incompressibility of fraction $1 - \phi_R^{max}$ of the proteome. The different slopes (Fig. 3e, 3h, S3g, S3i) we observe in *B. subtilis* suggest possible differences in the growth laws in this species.

Appendix References

Dai X, Zhu M, Warren M, Balakrishnan R, Patsalo V, Okano H, Williamson JR, Fredrick K, Wang YP & Hwa T (2016) Reduction of translating ribosomes enables Escherichia coli to maintain elongation rates during slow growth. *Nat Microbiol* 2: 1–9

Kearns DB, Chu F, Branda SS, Kolter R & Losick R (2005) A master regulator for biofilm formation by Bacillus subtilis. *Mol Microbiol* 55: 739–749

Mukherjee S & Kearns DB (2014) The Structure and Regulation of Flagella in Bacillus subtilis . *Annu Rev Genet* 48: 319–340

Murray EJ, Kiley TB & Stanley-Wall NR (2009) A pivotal role for the response regulator DegU in controlling multicellular behaviour. *Microbiology* 155: 1–8

Scott M, Gunderson CW, Mateescu EM, Zhang Z, Hwa T, Gunderson CW, Mateescu EM, Zhang Z & Hwa T (2010) Interdependence of Cell Growth Origins and Consequences. *Science (80-)* 330: 1099–1102

Scott M, Klumpp S, Mateescu EM & Hwa T (2014) Emergence of robust growth laws from optimal regulation of ribosome synthesis. *Mol Syst Biol* 10: 747

Shaw LB, Zia RKP & Lee KH (2003) Totally asymmetric exclusion process with extended objects: a model for protein synthesis. *Phys Rev E Stat Nonlin Soft Matter Phys* 68: 021910

Winkelman JT, Bree AC, Bate AR, Eichenberger P, Gourse RL & Kearns DB (2013) RemA is a DNA-binding protein that activates biofilm matrix gene expression in Bacillus subtilis. *Mol Microbiol* 88: 984–997

You C, Okano H, Hui S, Zhang Z, Kim M, Gunderson CW, Wang YP, Lenz P, Yan D & Hwa T (2013) Coordination of bacterial proteome with metabolism by cyclic AMP signalling. *Nature* 500: 301–306

Zhu M, Mori M, Hwa T & Dai X (2019) Disruption of transcription-translation coordination in Escherichia coli leads to premature transcriptional termination. *Nat Microbiol* 4: 2347–2356

APPENDIX FIGURES

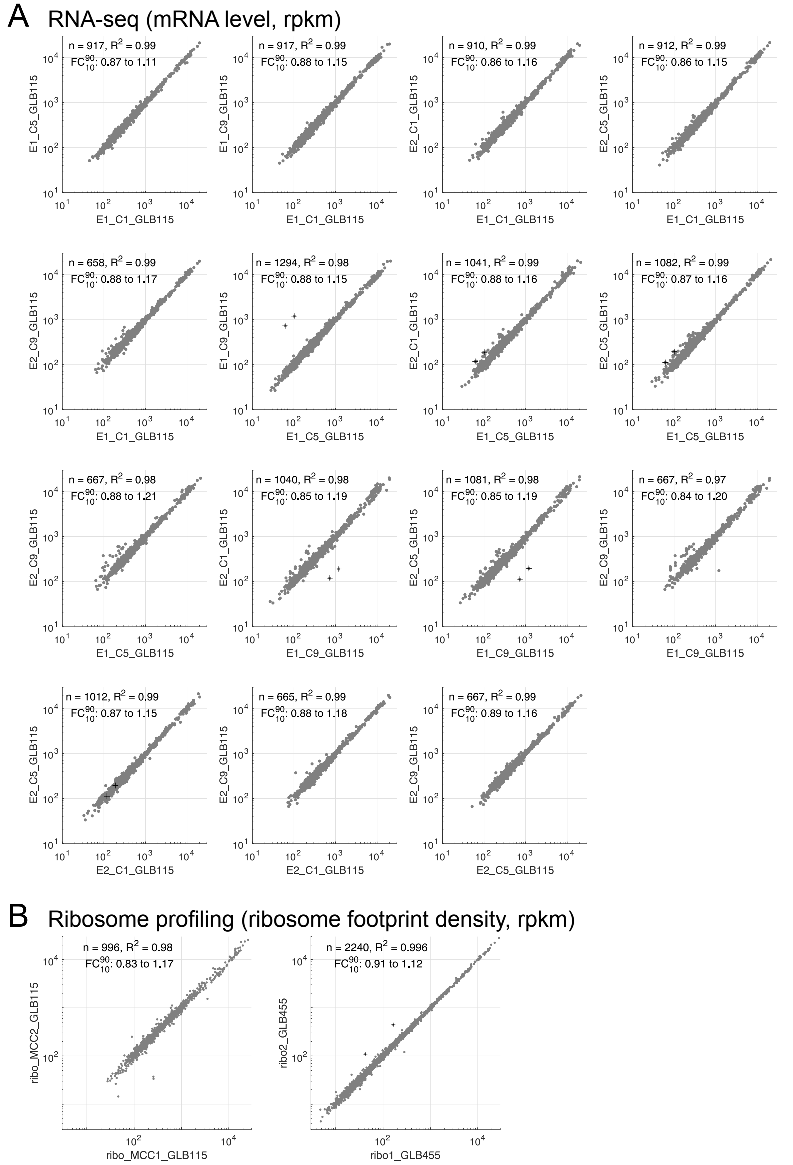

Appendix Figure S1 - Assessment of reproducibility of expression quantification.

A Pairwise comparison of mRNA level quantification for biological replicates (wild-type). Each plot shows the mRNA level quantification (units of rpkm) for all possible pairs across our 6 wild-type RNA-seq datasets, with the only difference being different inducer [xylose and/or IPTG, genes *xylA* and *xylB*, endogenously responsive to xylose, are marked by +] concentration. Axes label correspond to the sample names (E_C_strain, E: experiment number, C: conditions, see Dataset EV3 for descriptions). All genes with more than 5 reads mapped in each sample are shown (number of genes indicated). The number of genes with >100 reads mapped, the R^2 for the log-transformed mRNAs and 10th to 90th percentile in fold-change FC_{10}^{90} for each sample pair are shown.

B Pairwise comparison of ribosome profiling data for biological replicates from wild-type (GLB115) and strain GLB455 (*B. subtilis* with ectopic fluorescent proteins under IPTG and xylose promoters). Each sample pair was grown in the same batch of MCC medium with different inducer concentration (which should leave all but genes *xylA* and *xylB* [marked by +] endogenously responsive to xylose, unchanged). The number of genes with >100 reads mapped, the R^2 for the log-transformed mRNAs and 10th to 90th percentile in fold-change FC_{10}^{90} for each sample pair are shown.

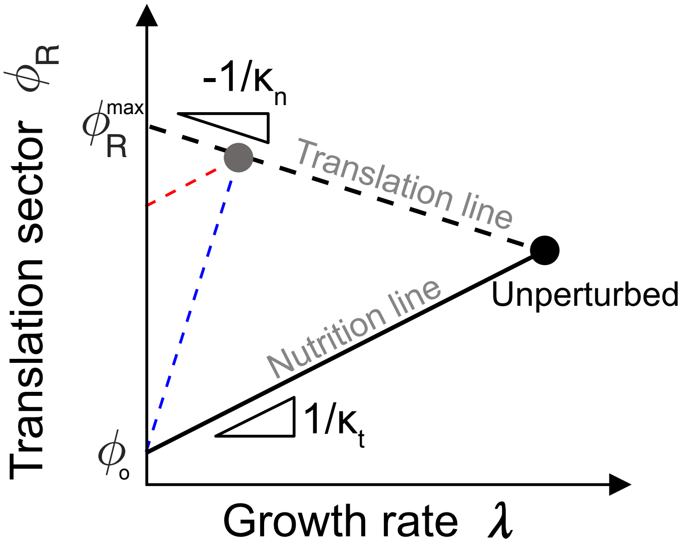

Appendix Figure S2 - Schematic summary of bacterial growth laws.

Translation sector (proteome fraction to translation proteins) vs. growth rate. Upon change in growth medium quality or overexpression of unnecessary non-toxic proteins, the cell's physiological state moves along $\phi_R = \phi_\circ + \frac{\lambda}{\kappa_t}$ (nutrition line). Following perturbation to translation, trajectories follow $\phi_R = \phi_R^{max} - \frac{\lambda}{\kappa_n}$ (translation line). Composition of nutritional and translation perturbation can in principle be used to identify nature of perturbation to translation (increase in inactive ribosome, red line vs. decrease in translational capacity, blue line).

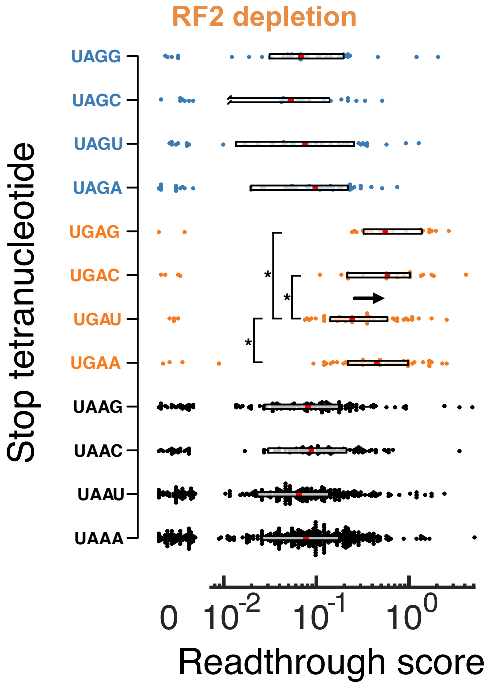

Appendix Figure S3 - Translation readthrough score under acute RF2 CRISPRi knockdown stratified by stop tetranucleotide.

Translation readthrough score (see Fig. 4B) for isolated genes (>0.1 ribosome footprint reads/nt) stratified by stop tetranucleotide for RF2 CRISPRi knock down. Points in beeswarm plot correspond to individual genes, with overlaid box plot highlighting the interquartile range (25th to 75th percentile, median red mark). Arrow points to the ≈2× difference between UGAC and UGAU. * indicates $p<0.05$, with significantly more readthrough for UGA[C/G/A] compared to UGAU (Wilcoxon rank-sum test on log transformed values, excluding zeros, UGAG vs. UGAU: $p = 0.0008$; UGAC vs. UGAU: $p = 0.006$; UGAA vs. UGAU: $p = 0.017$).

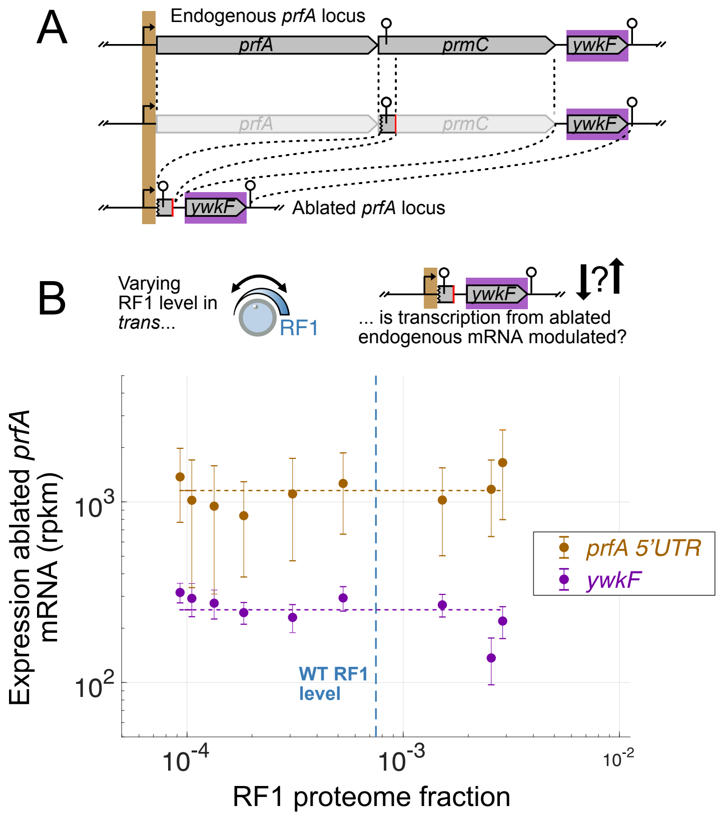

Appendix Figure S4 - Response of RF1 promoter to varying RF1 expression.

A Schematic illustration of the genetic changes made to the endogenous *prfA* (RF1) locus in orthogonally tunable RF1/PrmC strain GLB438. The open reading frame for *prfA* is completely removed, leaving the 5' UTR (brown) and promoter region unperturbed. The terminator intragenic to *prmC* is preserved (start codon of *prmC* removed, and in-frame stop added after the terminator). The *prmC/ywkF* intergenic region and open reading frame for *ywkF* is unchanged.

B Quantification of the RNA level of the 5' UTR (brown) and *ywkF* gene (purple) for the experimental series perturbing RF1 expression (experiment E2, Fig. 1A), showing no substantial changes in expression across the full range of RF1 expression tested. Error bars correspond to standard deviation of bootstrap resampling estimates to assess technical read counting noise from a single experiment.

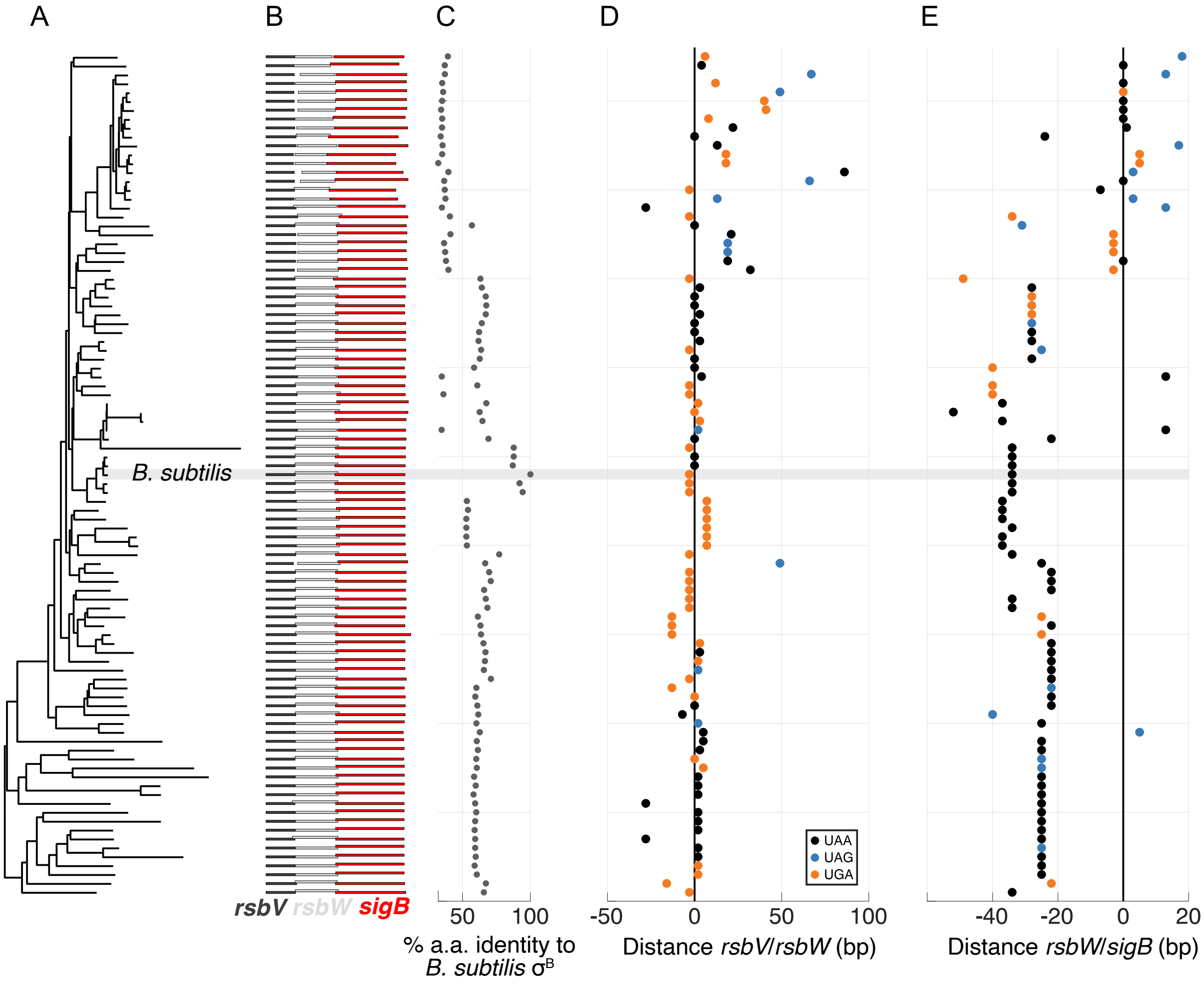

Appendix Figure S5 - σ^B operon conservation.

Bioinformatic analysis of σ^B operons homologous to that of *B. subtilis* (Methods).

A Phylogenetic tree (neighbor joining from consensus 16S rRNAs) for 95 species with candidate σ^B operons (Methods, full list in Dataset EV9).

B To-scale miniature representation of gene organization for σ^B operon candidates (dark gray: *rsbV* homolog, pale gray: *rsbW* homolog, red: *sigB* homolog).

C Percent identity (amino acid) to *B. subtilis'* σ^B.

D-E Distance between sequential genes in the operon, colored by stop codon of the upstream gene for **D** *rsbV/rsbW* and **E** *rsbW/sigB*. 18/95=19% candidate σ^B operons have the **AUGA** *rsbV/rsbW* overlap, compared to 226/3042=7% of all co-directional gene pairs in *B. subtilis*. 42/95=44% of the candidate operons have any form of coding sequence overlap (distance < 0 bp) between *rsbV* and *rsbW*, compared to 521/3024=17% across all co-directional gene pairs in *B. subtilis*.

Related to Fig. 5.
