# Supplementary material for: Spurious regulatory connections dictate the expression‐fitness landscape of translation factors
Source: Mol Syst Biol. 2021 Apr 26;17(4):e10302. doi: 10.15252/msb.202110302 (PMC8073009; doi:10.15252/msb.202110302)
Supplement: Supplementary file 2 — Expanded View Figures PDF [file MSB-17-e10302-s001.pdf]

Expanded View Figures

Figure EV1. Details of RF-inducible expression constructs.

A Schematic tunable expression system. Inducible constructs are added at safe harbor loci, together with a barcode for competition experiments. The endogenous gene copy is then deleted in a scarless fashion.

B, C Details of loci with tunable expression cassettes for the orthogonally tunable (B) RF2 and PrmC strain, and (C) RF1 and PrmC strain. Disrupted safe harbor endogenous loci are *amyE*, *lacA*, and *levB*. Control strains were also constructed with blank expression cassettes at these locations (Materials and Methods, Fig EV2F, G, I and J). Inducible repressors XylR and LacI, respectively, responsive to IPTG and xylose are shown in black. Resistance cassettes are shown in yellow. The location of the 8-nt chromosomal barcode in one arm of the *amyE* homology region is shown in purple.

D Fold-change in RF levels as a function of inducers, with fitted Hill curve, serves as a guide to the eye. Endogenous expression is shown as the horizontal dashed line (fold-change of 1) and the full attainable dynamic range indicated on the right. See Materials and Methods for calibration to proteome fraction.

E, F 3D RF expression space for all phenotypically profiled conditions (shown separately in Fig 2A–D) shown in (E), and orthogonal projection in 2D subspaces in (F). Dashed lines mark endogenous expression levels.

 © 2021 The Authors

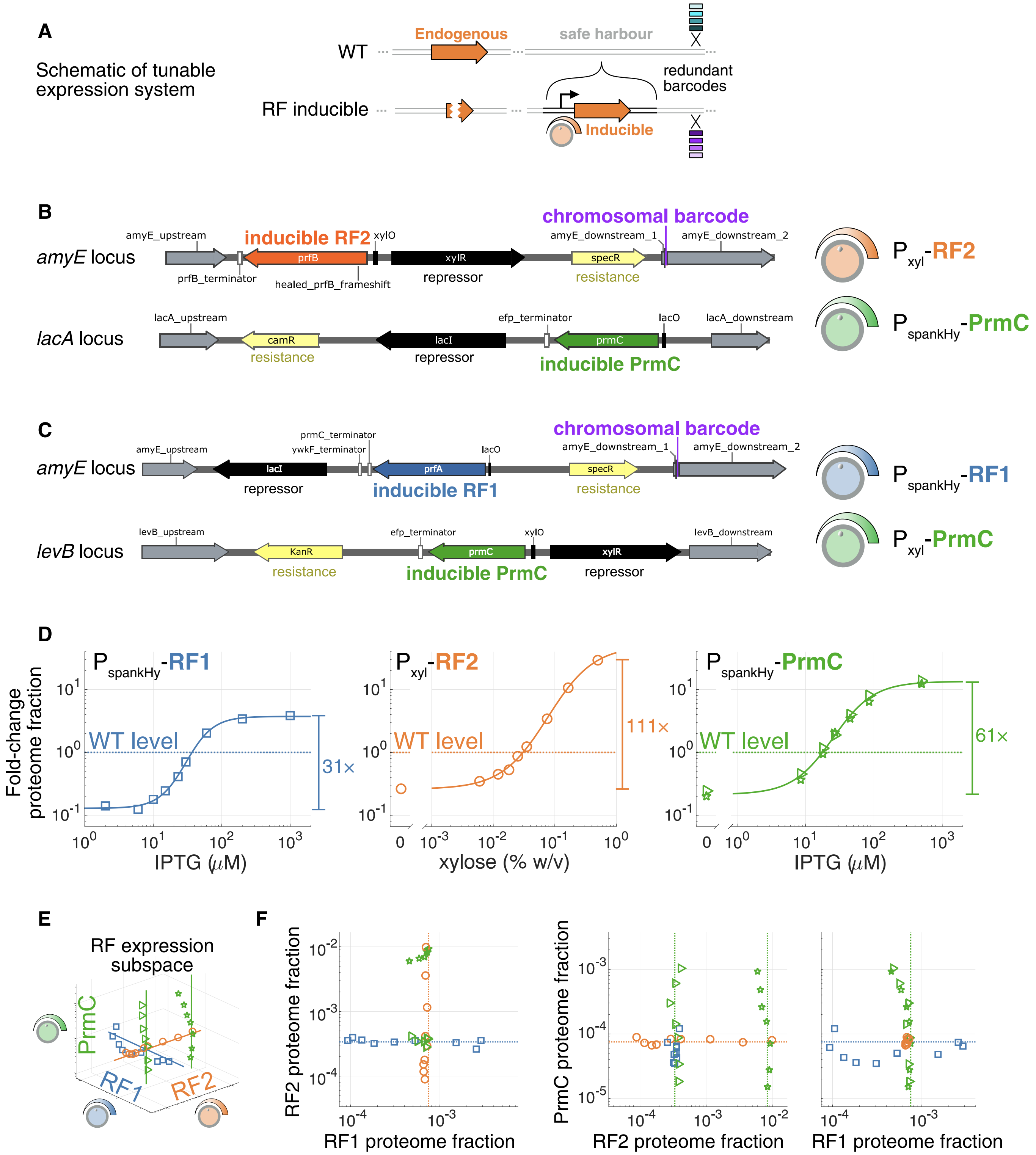
A
Schematic of tunable expression system
WT
Endogenous
safe harbour
redundant barcodes
RF inducible
Inducible
B
amyE locus
amyE_upstream
inducible RF2
xylO
chromosomal barcode
amyE_downstream_1
amyE_downstream_2
prfB
xylR
specR
prfB_terminator
healed_prfB_frameshift
repressor
resistance
P_{xyl}-RF2
lacA locus
lacA_upstream
efp_terminator
lacO
lacA_downstream
camR
lacI
prmC
resistance
repressor
inducible PrmC
$P_{spankHy}$-PrmC
C
amyE locus
amyE_upstream
prmC_terminator
ywkF_terminator
lacO
chromosomal barcode
amyE_downstream_1
amyE_downstream_2
lacI
prfA
specR
repressor
inducible RF1
resistance
$P_{spankHy}$-RF1
levB locus
levB_upstream
efp_terminator
xylO
levB_downstream
KanR
prmC
xylR
resistance
inducible PrmC
repressor
P_{xyl}-PrmC
D
Fold-change proteome fraction
$P_{spankHy}$-RF1
WT level
31×
IPTG (μM)
P_{xyl}-RF2
WT level
111×
xylose (% w/v)
$P_{spankHy}$-PrmC
WT level
61×
IPTG (μM)
E
RF expression subspace
PrmC
RF1
RF2
F
RF2 proteome fraction
RF1 proteome fraction
PrmC proteome fraction
RF2 proteome fraction
RF1 proteome fraction

Figure EV1.

Figure EV2. Details of relative growth rate measurement.

A Schematic of competition experiments. Pools of barcoded strains competed for ≈ 30 generations with five samplings, barcode frequencies are quantified, and changes in barcode frequencies over time determined. Relative growth rate is $\lambda_{\text{inducible}}/\lambda_{\text{WT}} = 1 + s$, where s is the slope of the $\log_2$ barcode ratio vs. time (number of generations). This process was performed for all induction conditions shown in Fig 2A–D.

B Schematic the barcode readout procedure, carried out in two PCR steps from genomic DNA extracted from pools of competing strains, with UMI and first index added at the first PCR, and a second index added at the second PCR. Details of the final amplicon for barcode readout are in Dataset EV6.

C, D Examples of barcode frequency ratios over time for isogenic strain pairs from a single competition experiment, (C) wild-type vs. wild-type, and (D) RF2 overexpression vs. wild-type. Representative strain pairs from experiment E1-C9 are shown. Inferred $s := \lambda_{\text{inducible}}/\lambda_{\text{WT}} - 1$ from the linear fit (black line) is shown on the graph. Range of slopes s_{min}–s_{max} of subsampled bootstraps (gray lines) is reported as $s^{s_{max}}_{s_{min}}$. Dashed lines correspond to 95% confidence interval. Error bars correspond to estimated noise attributable from Poisson counting noise in UMI counts ($\frac{1}{\log(2)}\sqrt{\frac{1}{N_1}+\frac{1}{N_2}}$, where N_1 and N_2 are the respective UMI barcode counts for the compared strain pairs at the corresponding time point).

E Distribution of measured s for pairs of strains with identical genotype apart from barcode across all experiments ($n = 1{,}253$ comparisons, e.g., 4/1,253 experimentally determined s are shown in panel C). The shaded gray area corresponds to the $\pm\, 2\sigma_s = \pm\, 1.2\%$ shown in Fig 2E–H.

F, G Measured fitness difference to wild-type for strains with blank expression cassettes. (F) Blank P_{xyl} at *amyE* & blank $P_{spankHy}$ at *lacA*, strains GLB434–437, $n = 644$ comparisons. (G) Blank $P_{spankHy}$ at *amyE* & blank P_{xyl} at *leuB*, strains GLB446–449, $n = 252$ comparisons. Both control strain series show minimal effect of ectopic insertions on cell fitness. s values displayed correspond to median with 25th and 75th percentile of values across isogenic pairs.

H Hand mixing experiment with two strains with different barcodes, showing accurate (slopes 1.00 and 0.98 for observed vs. expected ratio of barcodes from two technical replicates) readout of cell frequencies in pool over nearly four orders of magnitude. Median difference between readout from two technical replicates is 20%. Error bars are as in (C and D).

I, J Representative examples of mRNA level (RNA-seq) comparison between wild-type and control strains with blank expression cassette insertions: (I) GLB434 vs. wild-type (experiment E1-C5), and (J) GLB446 vs. wild-type (experiment E2-C1). Cumulative distributions of fold-changes are shown as insets as in Fig 3A and B.

 © 2021 The Authors

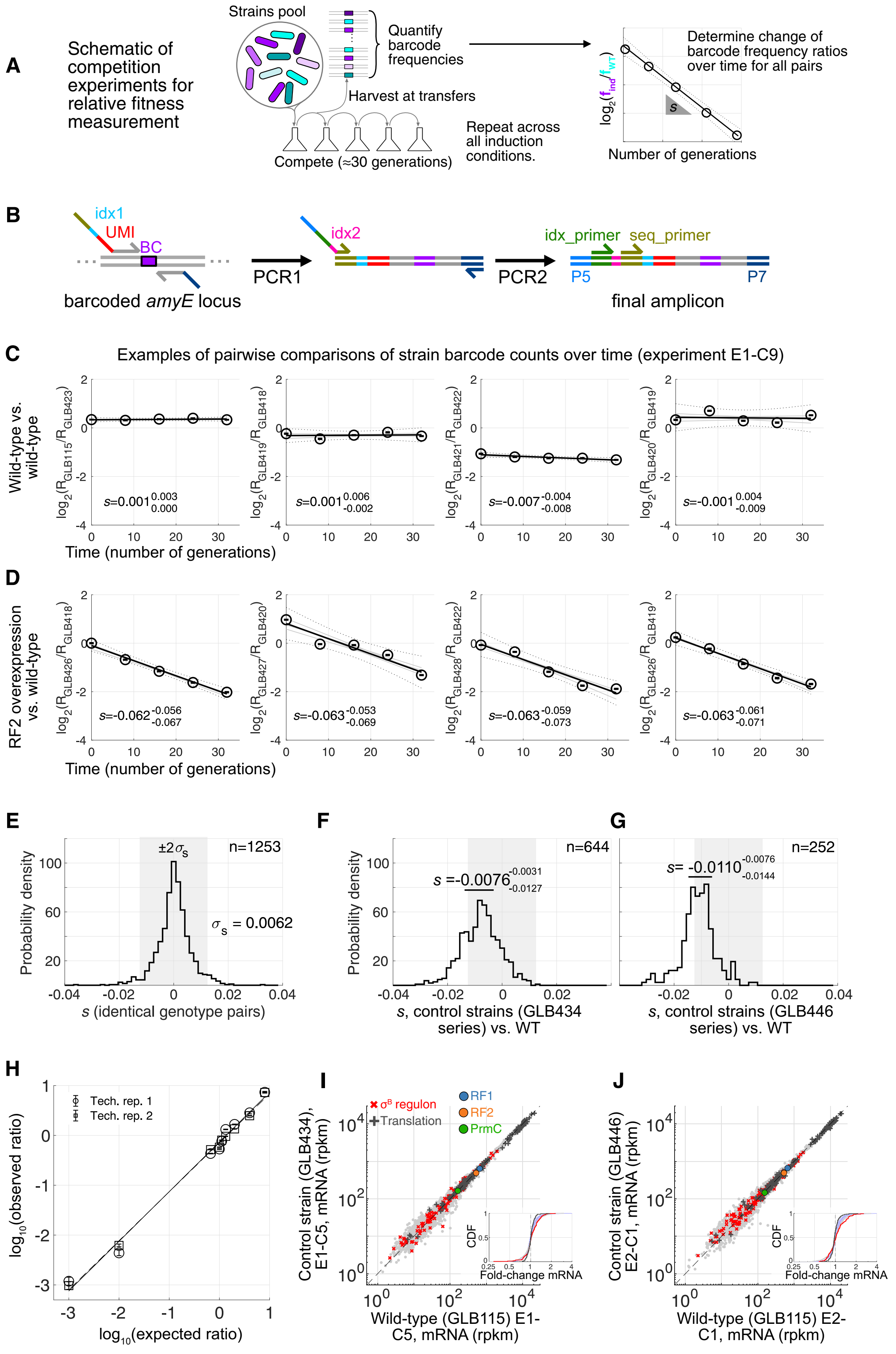
A
Schematic of competition experiments for relative fitness measurement
Strains pool
Quantify barcode frequencies
Harvest at transfers
Compete (≈30 generations)
Repeat across all induction conditions.
Determine change of barcode frequency ratios over time for all pairs
Number of generations
B
idx1
UMI
BC
barcoded amyE locus
PCR1
idx2
PCR2
idx_primer
seq_primer
P5
P7
final amplicon
C
Examples of pairwise comparisons of strain barcode counts over time (experiment E1-C9)
Wild-type vs. wild-type
Time (number of generations)
D
RF2 overexpression vs. wild-type
Time (number of generations)
E
n=1253
σs = 0.0062
Probability density
s (identical genotype pairs)
F
n=644
s, control strains (GLB434 series) vs. WT
G
n=252
s, control strains (GLB446 series) vs. WT
H
Tech. rep. 1
Tech. rep. 2
log10(observed ratio)
log10(expected ratio)
I
σB regulon
Translation
RF1
RF2
PrmC
Control strain (GLB434), E1-C5, mRNA (rpkm)
Wild-type (GLB115) E1-C5, mRNA (rpkm)
CDF
Fold-change mRNA
J
Control strain (GLB446) E2-C1, mRNA (rpkm)
Wild-type (GLB115) E2-C1, mRNA (rpkm)

Figure EV2.

Figure EV3. Interplay between expression of RF2 and PrmC, fitness and σ^B regulon activation.

A–I Analogous to Fig 3, but for varying PrmC levels in conjunction with RF2 overexpression (conditions shown in Fig 2D). In (E–H), open light green pentagrams correspond to cells with *sigB*, and filled dark green hexagrams to cells without *sigB* (deletion). Blue shadings mark the region of the expression space for which the growth defect is not rescued by *sigB* deletion, indicating a different underlying cause for the decrease in translation sector.

J Comparison of expression at maximal PrmC expression for endogenous and overexpressed RF2 levels (respective comparisons to unperturbed conditions in Fig 3A and current panel C), showing highly reproducible σ^B induction independent of RF2 levels (the two outliers marked by black circles are *xylA* and *xylB*, which are responsive to xylose).

K Expression-fitness landscape for RF2 and PrmC.

L–N orthogonal projections from K showing the (L) RF2, and (M, N) PrmC directions. Panel (N) is the PrmC fitness landscape, with the fitness defect caused by RF2 overexpression defect subtracted out (arrows in panels L and M). Fitness defect at overexpressed PrmC is independent of RF2 (dashed black line in N). Knockdown defect is exacerbated by RF2 overexpression (black arrows in N).

O, P Transcriptome under RF2 expression perturbation. (O) RF2 knockdown shows modest σ^B induction, whereas (P) maximal RF2 overexpression displays little expression changes.

Q Growth rate difference for strain with inducible RF2, with and without *sigB*. A mild but significant ($P < 10^{-5}$, bootstrap subsampling, Materials and Methods) improvement in fitness upon *sigB* deletion at lowest RF2 levels is seen. Measured *s* for each of 12 strain pairs, inducible RF2 (GLB426 to GLB429) vs. inducible RF2 without *sigB* (GLB430 to GLB433), are shown for all profiled RF2 levels, with the median and 25th to 75th percentile marked by black lines (error bars). Red marks the condition with lowest RF2 level. Inset shows cumulative distribution of relative fitness difference for lowest RF2 level (red) and rest of conditions (black). The fitness rescue upon *sigB* deletion (median increase in fitness $\Delta s = 0.009$) is commensurate with the estimated excess σ^B regulon proteome fraction ($\Delta\phi_{SigB} = 0.0085$) in this condition.

 © 2021 The Authors

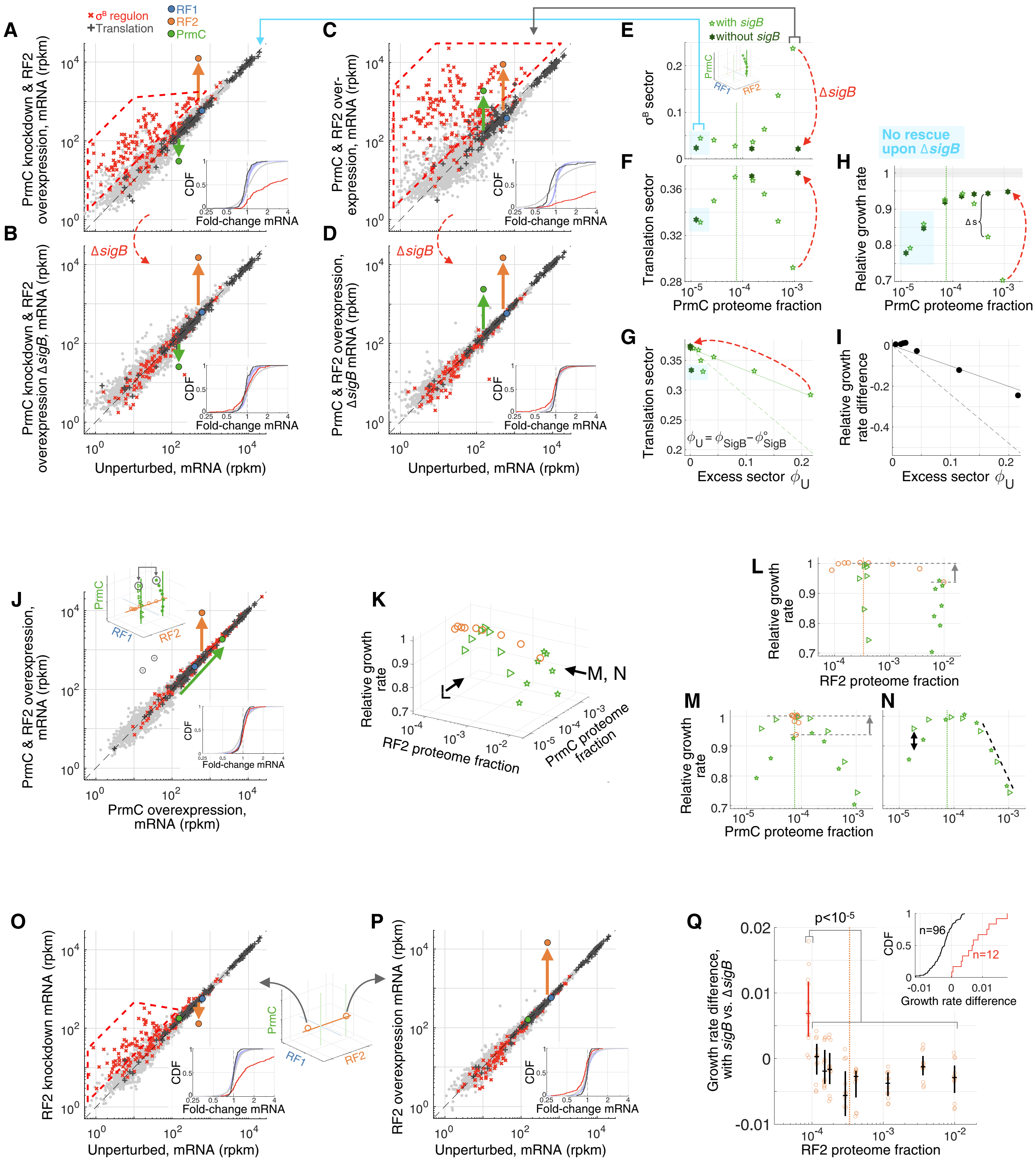
A
B
C
D
E
F
G
H
I
J
K
L
M
N
O
P
Q
σB regulon
Translation
RF1
RF2
PrmC
with sigB
without sigB
ΔsigB
No rescue upon ΔsigB
PrmC knockdown & RF2 overexpression, mRNA (rpkm)
PrmC knockdown & RF2 overexpression ΔsigB, mRNA (rpkm)
PrmC & RF2 over-expression, mRNA (rpkm)
PrmC & RF2 overexpression, ΔsigB mRNA (rpkm)
Unperturbed, mRNA (rpkm)
Fold-change mRNA
CDF
σB sector
Translation sector
PrmC proteome fraction
Relative growth rate
Excess sector ϕU
Relative growth rate difference
ϕU = ϕSigB − ϕ°SigB
Δs
PrmC & RF2 overexpression, mRNA (rpkm)
PrmC overexpression, mRNA (rpkm)
RF2 proteome fraction
M, N
RF2 knockdown mRNA (rpkm)
RF2 overexpression mRNA (rpkm)
Growth rate difference, with sigB vs. ΔsigB
p<10-5
n=96
n=12
Growth rate difference

Figure EV3.

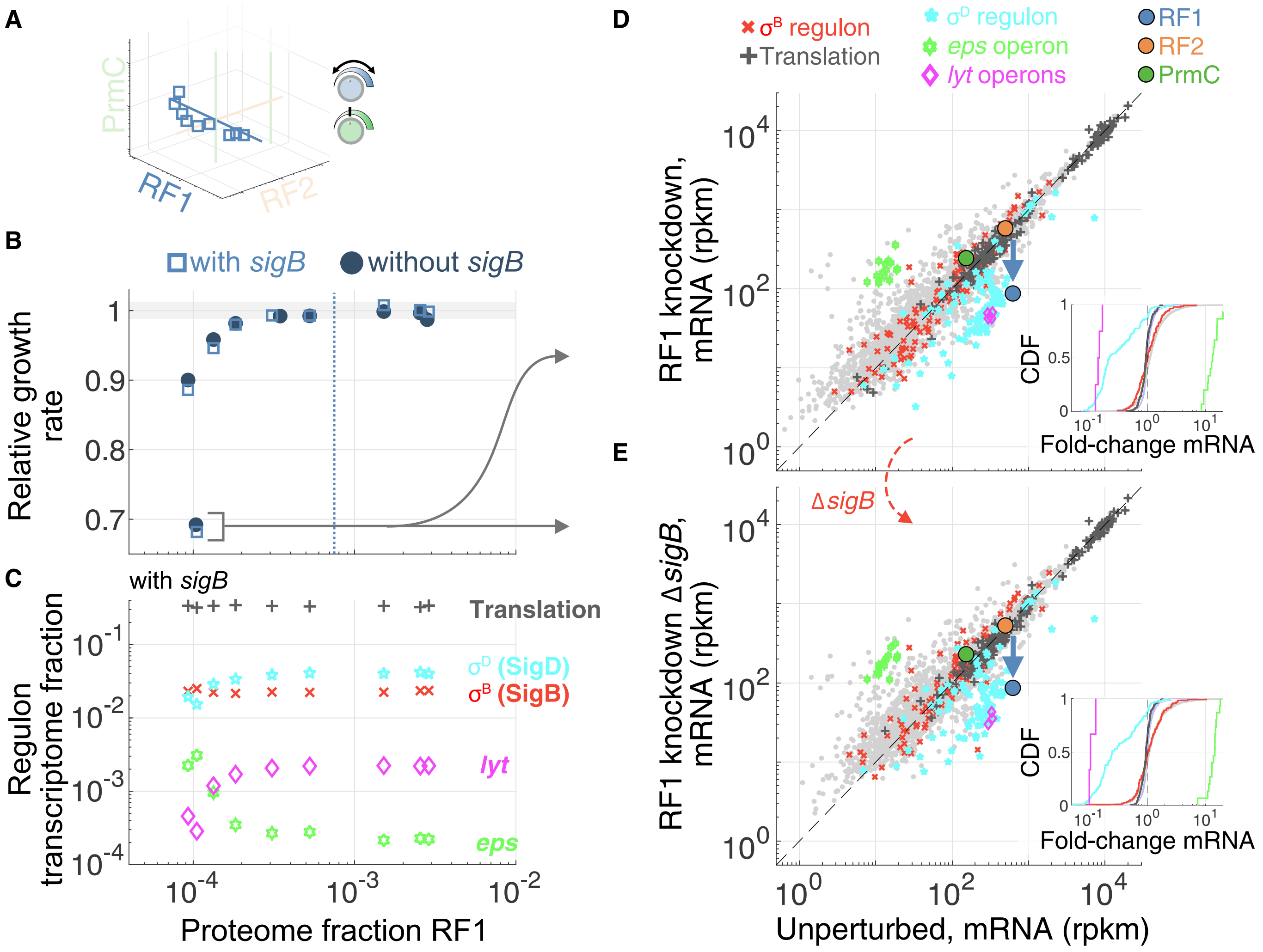

Figure EV4. Transcriptomics changes upon RF1 knockdown.

A Profiled RF1 levels in the RF expression subspace (reproduction of Fig 2A).

B Fitness defect upon modulation of RF1 level, with (open pale blue squares) and without *sigB* (filled dark blue circles), showing no strong influence of σ^B.

C Quantification of various regulons' transcriptome fraction as a function of RF1 levels (shown for strain with *sigB*). The large fitness decrease upon RF1 knockdown coincides with decrease in motility (SigD, cyan) regulon and autolysin operon (magenta), and increase in biofilm matrix *eps* (light green) genes production.

D, E mRNA levels (rpkm, genes with > 5 reads mapped shown) comparison to unperturbed for maximal RF1 knockdown, with regulon members colored following (c), for strains (D) with or (E) without *sigB*. Median fold-change is larger or equal to 5 for SigD, *eps*, and *lyt* genes, independently from σ^B. Cumulative distributions of fold-change for highlighted regulons (rest of genes in gray, all-to-all for across unperturbed replicates in pale blue) are shown as insets as in Fig 3A.

 © 2021 The Authors

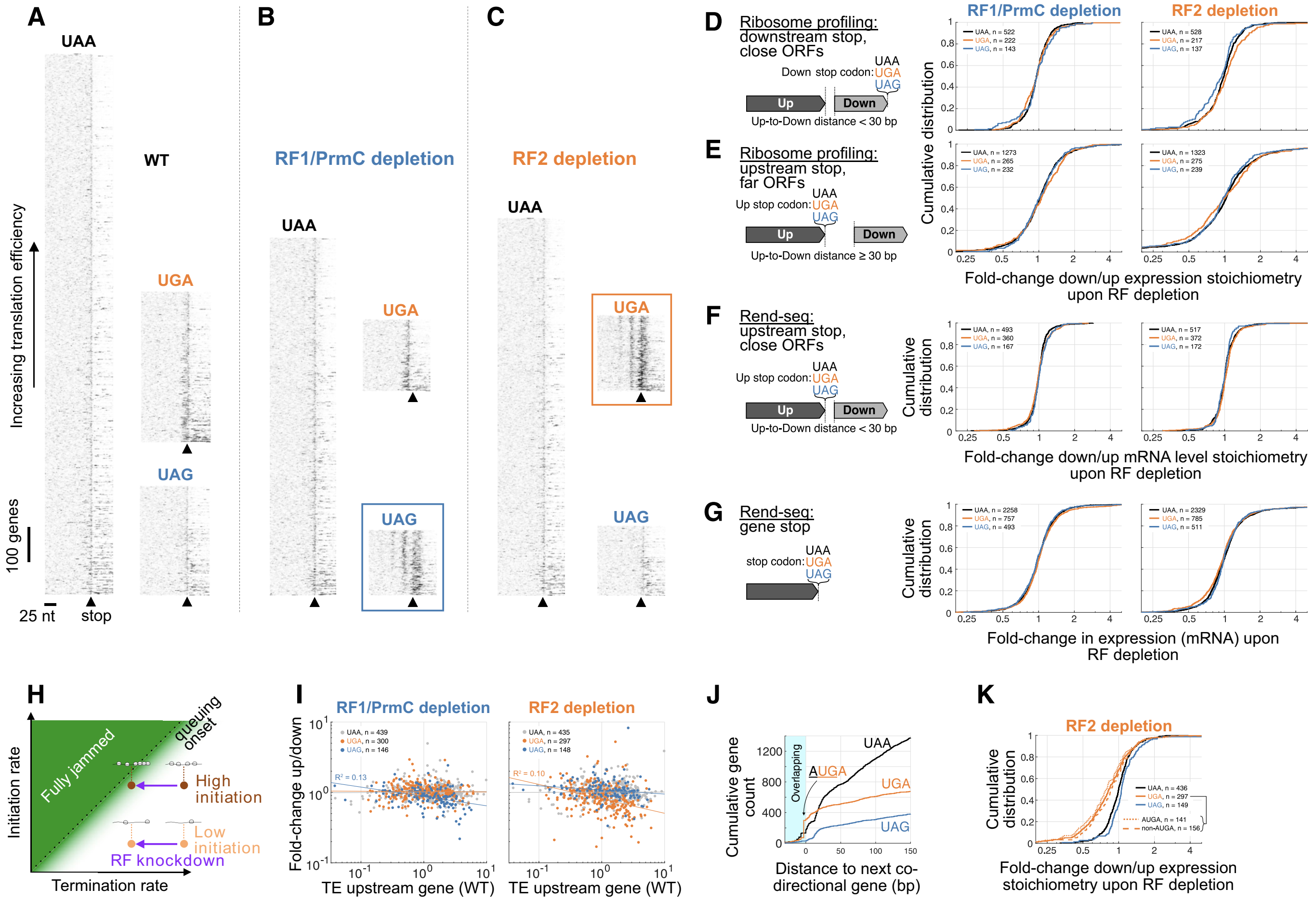

Figure EV5. Details on translational profiling following acute RF depletion.

A–C Heatmaps of all data used to generate metagene ribosome queuing plot shown in Fig 4A for (A) wild-type, (B) RF1/PrmC CRISPRi depletion, and (C) RF2 CRISPRi depletion. Genes are separated by stop codon. Each horizontal line represents a gene, and gene-normalized ribosome footprint density (center-mapped) is shown as gray scale, horizontally aligned by the position of the stop codon (5′–3′ left to right). Genes are organized in increasing order of translation efficiency moving up (TE). Queues upstream of stop codons with perturbed RFs can be seen (colored boxes in B and C) and are longer for genes with high TE. Scale bars indicate 25 nt and 100 genes. Caret ▲ marks the position of stop codons.

D Control analysis of changes in expression stoichiometry under RF depletion for co-directional genes within 30 bp, but stratified by the stop codon of the downstream gene (RF1/PrmC: $FC_{UAG} = 0.99$, $P = 0.39$; RF2: $FC_{UGA} = 0.98$, $P = 0.27$, P-value from stop codon reshufflings, Materials and Methods).

E Similar to (D), but for co-directional genes separated by more than 30 bp, stratifying by the upstream gene (RF1/PrmC: $FC_{UAG} = 1.01$, $P = 0.60$; RF2: $FC_{UGA} = 1.04$, $P = 0.96$, P-value from stop codon reshufflings, Materials and Methods).

F Analysis for expression stoichiometry of gene pairs paralleling Fig 4D, but with Rend-seq data, showing no effect (RF1/PrmC: $FC_{UAG} = 1.00$, $P = 0.59$; RF2: $FC_{UGA} = 1.02$, $P = 0.98$, P-value from stop codon reshufflings, Materials and Methods). This further suggests that perturbed expression stoichiometry results from changes in translation.

G Distributions of fold-change in mRNA levels between wild-type and RF depletion stratified by stop codon, showing small (≈ 2%) changes in median for RF-perturbed stop (RF1/PrmC: $FC_{UAG} = 0.98$, $P = 0.06$; RF2: $FC_{UGA} = 0.98$, $P = 0.02$, P-value from stop codon reshufflings, Materials and Methods).

H Schematic illustrating how decreasing termination rate leads to ribosome queues on mRNAs with high translation efficiency (ribosome initiation rate), see Appendix Supplementary Methods.

I Fold-change in expression stoichiometry for gene pairs considered in Fig 4E (within 30 bp and stratified by upstream stop codon, subset with measured TE shown) as a function of TE. Significant (F-test from MATLAB's regress function, $P < 0.05$) correlations (increasing translation efficiency leading to more severe effect, $R^2 \approx 0.1$) are seen for genes with stop cognate to the RF perturbation.

J Cumulative number of co-directional gene pairs separated by given distance in *Bacillus subtilis*, stratified by stop codon identity. Overlap **AUGA** (arrow) is the most common configuration.

K Same as Fig 4D, but with UGA pairs split between those with **AUGA** overlap or not, with the overall effect distribution is similar between the two types of overlaps.
